# Successful hemostasis of colonic diverticular bleeding using single-balloon-assisted endoscopy and endoscopic band ligation in a patient with difficult total colonoscopy

**DOI:** 10.1055/a-2512-0667

**Published:** 2025-01-21

**Authors:** Takaaki Kishino, Takayuki Sawa, Toshihito Tsuchihashi

**Affiliations:** 1 53356 Department of Gastroenterology and Hepatology, Center for Digestive and Liver Diseases, Nara City Hospital, Nara, Japan

Recently, cases of colonic diverticular bleeding (CDB) in elderly individuals have significantly increased 1 . Effective management of CDB is crucial, as severe cases may require transarterial embolization (TAE) or surgery, which can be life-threatening for elderly patients with multiple comorbidities 2 . Given the invasiveness of surgery and TAE, minimally invasive treatments, such as endoscopic hemostasis, are preferable, especially for elderly patients. However, traditional endoscopic methods may sometimes fail to reach the bleeding site, necessitating alternative approaches. Here, we report a case of recurrent CDB in a 91-year-old woman who was treated successfully by a novel approach using a single-balloon-assisted endoscopy and endoscopic band ligation.

The patient initially presented with hematochezia due to CDB in the ascending colon. Endoscopic hemostasis was attempted, but the colonoscope could not be inserted into the bleeding site, so TAE was performed. Three months later, she was readmitted with massive hematochezia and signs of hemorrhagic shock. Contrast-enhanced computed tomography revealed extravasation in the ascending colon ( Fig. 1 ). Given the previous difficulty in total colonoscopy, we opted to use single-balloon endoscopy to facilitate the insertion of the colonoscope into the bleeding site. A band ligation device was attached onto the endoscope to enable endoscopic hemostasis on the spot once the bleeding site was identified ( Fig. 2 , Fig. 3 ). This approach allowed us to reach the bleeding diverticulum in the ascending colon, where we successfully performed band ligation to stop the bleeding ( Fig. 4 , Fig. 5 ; Video 1 ).

**Fig. 1:**
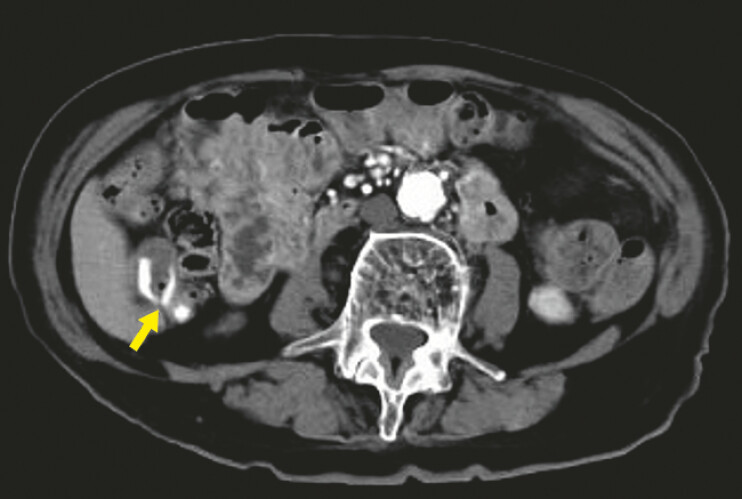
Contrast-enhanced computed tomography revealed extravasation in the ascending colon (arrow).

**Fig. 2:**
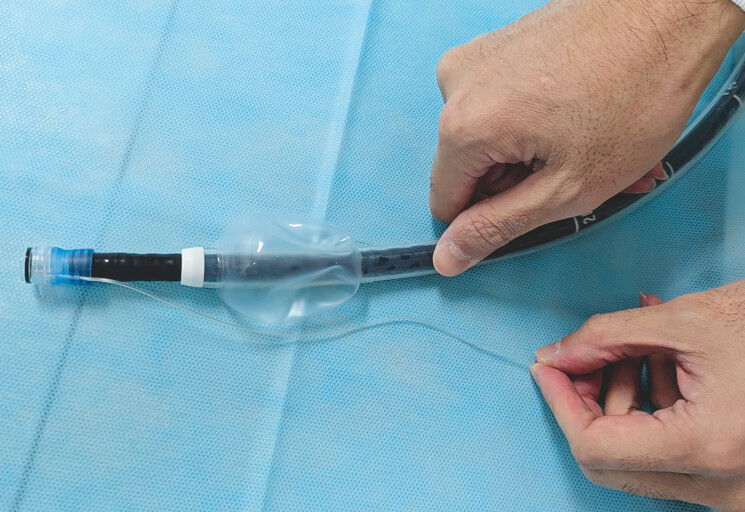
Devices used in endoscopic band ligation via single-balloon endoscopy. The colonoscope (PCF-PQ260L; Olympus, Tokyo, Japan) had an outer diameter of 9.2 mm, an effective length of 1680 mm, and no water-jet function. A band ligation device (MD-48709U; SB-Kawasumi Laboratories, Inc., Kanagawa, Japan) and a disposable sliding tube with a balloon (ST-SB1, Olympus) were attached to the endoscope.

**Fig. 3:**
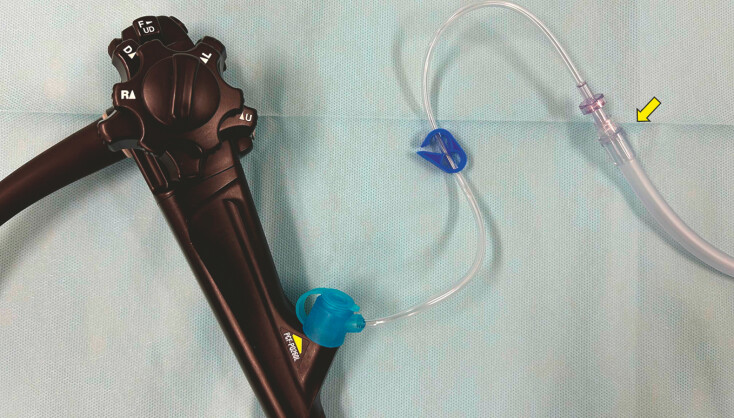
Devices used to clean the colon during colonoscopy. A water jet pump was connected to the irrigator (BioShield irrigator; US Endoscopy, Mentor, Ohio, USA) (arrow).

**Fig. 4:**
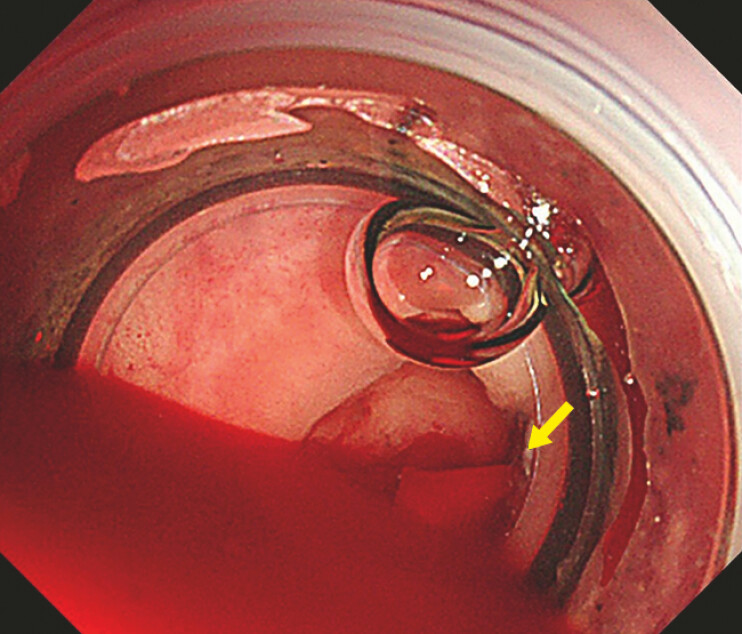
Endoscopy using underwater observation showed a diverticulum with active bleeding (arrow).

**Fig. 5:**
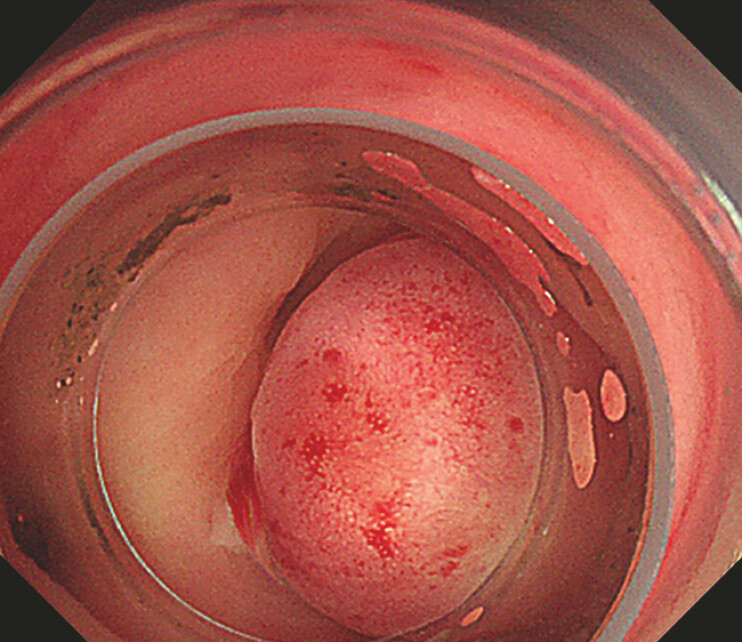
Colonic diverticulum treated with endoscopic band ligation.

Endoscopic hemostasis of colonic diverticular bleeding using single-balloon endoscopy and endoscopic band ligation in a patient with difficult total colonoscopy.Video 1

After treatment, the patient experienced no rebleeding or complications and was discharged in stable condition. This case demonstrates the effectiveness of balloon-assisted endoscopy and endoscopic band ligation in managing CDB with difficulty in total colonoscopy, offering an alternative to more invasive treatments in frail and elderly patients.

Endoscopy_UCTN_Code_TTT_1AQ_2AZ

## Notes

Endoscopy E-Videos https://eref.thieme.de/e-videos
*E-Videos* is an open access online section of the journal *Endoscopy* , reporting on interesting cases and new techniques in gastroenterological endoscopy. All papers include a high-quality video and are published with a Creative Commons CC-BY license. Endoscopy E-Videos qualify for HINARI discounts and waivers and eligibility is automatically checked during the submission process. We grant 100% waivers to articles whose corresponding authors are based in Group A countries and 50% waivers to those who are based in Group B countries as classified by Research4Life (see: https://www.research4life.org/access/eligibility/ ). This section has its own submission website at https://mc.manuscriptcentral.com/e-videos .
